# The Effect of Environmental Enrichment on Selected Physiological and Immunological Stress-Related Markers in Dairy Goats

**DOI:** 10.3390/biology13110859

**Published:** 2024-10-24

**Authors:** Yossi Wein, Ofri Vaidenfeld, Chris Sabastian, Enav Bar Shira, Sameer J. Mabjeesh, Haim Tagari, Aharon Friedman

**Affiliations:** Department of Animal Sciences, R.H. Smith Faculty of Agriculture, Food and Environment, The Hebrew University of Jerusalem, P.O. Box 12, Rehovot 7610001, Israel

**Keywords:** dairy goats, husbandry stress, environmental enrichment, advanced glycation end products, peripheral blood leukocytes, transferrin, oxytocin, serotonin

## Abstract

Homeostasis preservation is essential for animal survival, and any event that causes a disturbance in homeostasis is defined as a stressor. Here, we aimed to evaluate the effect of scratch brushes and stages as an environmental enrichment to alleviate stress in dairy goats. Twenty-four mixed-breed goats were divided into two groups according to common physiological conditions in breeding farms: milking and dry (milk-producing and non-milk-producing, respectively). Ten days after exposure to environmental enrichment treatment or not (control), blood was sampled. Following the enrichment, we observed a reduction in reactive oxidative stress metabolites, advanced glycation end products (AGEs), and their binding protein (transferrin) in the dry goats, as determined by an ELISA. In contrast, no change in AGEs, along with an increase in transferrin levels, was observed in the milking goats. Moreover, oxytocin levels decreased in the dry and increased in the milking goats, while serotonin levels increased in the dry and remained unchanged in the milking goats. Additionally, gene expression of the cytokines, IL-6 and IL-1ß, and anti-oxidative proteins, lysozyme and transferrin (in peripheral blood leukocytes), as determined by qPCR, presented the same pattern: down-regulation in the dry or up-regulation in the milking goats. In conclusion, a reliable methodology was developed for measuring husbandry stress in goats and to improve dairy goats’ husbandry practice. Current environmental enrichment produced different responsiveness in goats correlated to their physiological status: beneficial effect in dry goats, detrimental effect in milking goats.

## Simple Summary

Physiological equilibrium preservation is essential for an animal’s survival, and any event that may disturb this equilibrium is defined as a stressor. Here, we aimed to evaluate the effect of scratch brushes and stages as an environmental enrichment to reduce stress in dairy goats. Twenty-four mixed-breed goats were divided into two groups according to common physiological conditions in breeding farms: milking and dry (milk-producing and non-milk-producing, respectively). Blood was sampled ten days post-exposure to enrichment treatment or not (control). Following the enrichment, we observed a reduction in dry goats’ oxidative stress products and their binding protein, transferrin. In contrast, no change in these products, along with an increase in transferrin levels, was observed in milking goats. Moreover, the anti-stress hormones, oxytocin and serotonin, levels changed differentially between the dry- and milking-goat groups. Additionally, gene expression of immune-related and antioxidant molecules in white blood cells isolated from the goats’ blood presented the same pattern: down-regulation in dry or up-regulation in milking goats. In conclusion, a reliable methodology was developed for measuring husbandry stress in goats. Current environmental enrichment produced different responsiveness in goats correlated to their physiological status: beneficial effect in dry goats, detrimental effect in milking goats.

## 1. Introduction

Homeostasis is the physiological equilibrium state in which all animals strive to be. Accordingly, any factor that alters homeostasis is considered a stressor [1,2].

In order to meet the increased need to provide high-standard nutritional sources to the constantly growing world population, farming husbandry has shifted from extensive to intensive, resulting in more frequent exposure to stressors [3,4,5]. While stressors can be categorized according to their nature (physiological vs. mental) or origin (exogenous vs. endogenous), they all share a common ground: the potential to perturb homeostasis, reduce productivity, and, finally, the ability to induce morbidity and mortality [2,5,6].

Contemporary dairy goat husbandry practiced in most developed countries can serve as a good demonstration of this. As goats are (by nature) very rustic and herd animals, living in groups in the pasture, their relocation into intensive farming was found to have a deleterious effect on both physiological and mental homeostasis, as recent research suggests. This effect is frequently explained by exposure to more chronic stressors, such as narrowing the habitat environment and limiting available resources (pens vs. pasture), along with the exposure to more acute and stressful standard husbandry practices, such as transportation from one farm to another, insemination, handling, vaccination, and change in group arrangement; meaning, potential stressors can be of both a physiological and mental/social nature [7,8,9,10,11]. Additionally, it has been argued that the relocation of goats, which are a hierarchical species dominated by the higher female status in the group, into small and closed groups lacking in environmental enrichments and stimuli can further result in social stress, manifested by boredom, competition between individuals, and elevated aggressiveness of dominant goats toward low-status individuals in the group [7,8,9,11,12,13,14,15,16].

While it is intuitive that determining stress in farm animals is important for enhancing animal performance (fertility, growth rate, milk production, etc.) and farmer profitability so that resources are channeled for production instead of coping with stressors, it is also important for improving animal welfare, as it represents the way we look after the animals that serve us [1,4,5,8,9,17].

Eventually, homeostasis restoration success depends on the animal body’s ability to recruit sufficient physiological resources to cope with the perturbing stressors, along with an emphasis on the interaction between three main physiological networks: neural, endocrine, and immune systems. This is achieved using unique communication molecules, i.e., neurotransmitters, hormones, and cytokines [1,2,18,19,20,21,22,23,24]. Moreover, it has been suggested (by us and others) that oxidative stress serves as a common ground in many stress scenarios, and the measurement of its resulting reactive metabolites—advanced glycation end products (AGEs)—can serve as a reliable and sensitive oxidative stress marker in mammals as well as in birds. The immune system plays a considerable role in sensing this danger-associated molecular pattern (DAMP) and neutralizing it by increasing endogenous antioxidant reagents, such as transferrin and lysozyme and evoking a pro-inflammatory state [25,26,27,28,29,30].

Unfortunately, research on social stress and its effect on physiological and immunological parameters in modern-intensive goat farming are lacking. However, several recent papers suggest that environmental enrichment can alleviate husbandry-related stress in farm animals [31]. Aschwanden et al. suggested that goats’ environmental enrichment positively affected eating, resting, and aggressive behavior [32]. Similarly, Mandel et al. suggested that using scratch brushes as environmental enrichment in dairy cows has the potential to alleviate social stress and boredom since they serve as a source of interest for them [33].

Accordingly, in the current manuscript, we were prompted to explore the effect of environmental enrichment, in the form of scratch brushes and stages, on physiological, immunological, and stress-related parameters in dairy goats raised in intensive-farming systems. We hypothesized that this enrichment would have a beneficial effect on the goats’ homeostasis. However, as goats in different physiological statuses (dry vs. milking) may exhibit different physiological parameter sets, they also may exhibit different responses to the proposed enrichment. Thus, we were encouraged to investigate this response in each one of the physiological statuses separately.

## 2. Materials and Methods

### 2.1. Animals and Husbandry

Twenty-four 3–6-year-old, healthy Israeli Saanen cross-bred with local Nubian goats, non-pregnant dairy goats weighing 64.4 ± 2.64 kg (mean ± SEM), were reared in an enclosed, environmentally controlled goat facility (The Robert H. Smith Faculty of Agriculture, Food, and Environment, Rehovot, Israel). In accordance with a standard rearing protocol (Sheep and Goats Section, Ministry of Agriculture, Israel), the goats were fed a complete goat ration (adapted to their physiological status), and freshwater was provided *ad libitum* [34]. Goats were kept under natural light and dark cycles. The facility was equipped with commercial fans that turned on automatically when the ambient temperature reached 25 °C.

### 2.2. Ethics Statement

All goat studies were performed under an Institutional Animal Care and Use Committee-approved protocol of the Hebrew University of Jerusalem in compliance with Animal Welfare regulations (Approval no. AG-16589).

### 2.3. Environmental Enrichment

As the responsiveness of the goats to the environmental enrichment could be physiological-status-dependent, goats were divided accordingly into two commercially prevalent groups: “milking” and “dry” (milk-producing and non-milk-producing, respectively, *n* = 12 in each group). Goats in the milking groups were milked once daily by an automatic milking pallor (Afigoat, Afikim, Israel). The milk production averaged 2.14 ± 0.14 kg/day. The experiment started with randomly relocating the goats from each group into four identical 16 m^2^ yards (in each yard, *n* = 6). To allow the goats to habituate with each other and to the new environment, a 30-day adaptation period was provided, as recommended by Fernández et al. [12]. Following this period, each one of the groups (yards) in each of the physiological statuses either received environmental enrichment or not (control). Note that the control setup was exactly as the environmentally enriched one, only without the following described enrichments: Environmental enrichment was in the form of free access to an installed in-yard static scratch brush (length 80 mm; diameter 40 mm with the ability to swing from east to west and north to south at a 270° angle [Melasty^®^, Nïlüfer/Bursa, Türkey]) and a wooden stage (custom made from oak wood: 120 × 120 mm^2^ face area raised on wooden legs 80 mm high), as presented in Figure S1 in Supplementary Materials. The space beneath it functioned as a shelter for resting or hiding and the top as a playground and increase in the living area. Enrichment continued for 10 days, and then the groups (in each of the physiological statuses) were switched for an additional 10 days, meaning that for each of the physiological statuses and groups (yards), each of the goats received both control and environmental treatments.

### 2.4. Blood Collection

Blood was collected from each goat in each group on the last day of each treatment (day 10). Goats were manually restrained, and 7 mL of blood was withdrawn by venipuncture of the vena jugularis. The collected blood was rapidly distributed into different tubes: for serological assays and blood biochemistry, 2 mL was placed in Vacuette^®^ Z Serum Sep Clot Activator (Greiner Bio-one, KremsmÜnster, Austria) tubes. For complete blood count (CBC) and peripheral mononuclear leukocytes (PBMCs) isolation (followed by mRNA extraction), 5 mL of blood was placed in Vacuette^®^ K_3_EDTA (Greiner Bio-one, KremsmÜnster, Austria) tubes.

### 2.5. Serum Preparation

Following blood clot formation, the tubes were centrifuged at 2500× *g* for 5 min at room temperature; the serum was transferred into fresh pre-marked tubes and stored at −20 °C until further analysis.

### 2.6. Complete Blood Count (CBC) and Blood Biochemistry Analysis

The entire analysis was conducted by the diagnostic laboratory of the Hebrew University Veterinary Teaching Hospital (Rishon-Lezion, Israel). CBC samples were analyzed using an Advia 120 analyzer (Siemens, Erfurt, Germany), and the serum samples were analyzed using a Cobas Integra 400 Plus analyzer (Roche, Mannheim, Germany).

### 2.7. Blood Chemistry

#### 2.7.1. Determination of Serum Transferrin and AGE by ELISA

Transferrin and AGE levels were determined in serum samples by direct and indirect ELISA, respectively. Briefly, diluted serum samples were placed on ELISA plates. Serial dilutions in PBS (pH = 7.4) of AGE (Abcam, Waltham, MA, USA) were used as respective standards. Coated plates were incubated in a humidified chamber at 4 °C overnight and were then blocked using 0.5% skim milk (BD Biosciences, Difco, Franklin Lakes, NJ, USA) in PBS. Detection was performed using HRP-conjugated polyclonal rabbit anti-sheep transferrin IgG (Biorbyt, Cambridge, UK) or polyclonal rabbit anti-AGE IgG (Abcam, Waltham, MA, USA) and HRP-conjugated polyclonal goat anti-rabbit IgG H+L (Jackson Laboratories Inc., West Grove, PA, USA). TMB (Kirkegaard and Perry Laboratories, Gaithersburg, MD, USA) was used as substrate. Optical absorbance was determined at 450 nm using a Bio Tek microplate reader (Bio Tek, Winooski, VT, USA). To determine AGE levels in the serum, the sample’ absorbance values were compared to a standard curve formed from purified AGE.

#### 2.7.2. Determination of Oxytocin (Serum) and Serotonin (Serum) Levels by ELISA

Serum oxytocin and serum serotonin levels were determined using a general oxytocin ELISA kit (My BioSource, San Diego, CA, USA) or a general serotonin ELISA kit (ELK biotech, Denver, CO, USA), respectively. The procedures were performed according to the manufacturer’s instructions.

#### 2.7.3. Peripheral Blood Mononuclear Leukocyte Isolation

Isolation of PBMC was performed by layering 4 mL of K_3_EDTA-treated blood over 4 mL of histopaque 1083 (Sigma-Aldrich Inc., St. Louis, MO, USA) in pre-prepared fresh 15 mL conical tubes. Following centrifugation (800× *g*, 15 min, at room temperature), cells within the buffy coat (lymphocytes and monocytes) were carefully aspirated using a Pasteur pipette and transferred into a fresh tube. After that, cells were washed using PBS and counted, and trypan blue vitality was found to be >95%. Finally, cells were pelleted, and RNAzol^®^ RT (Molecular Research Center Inc., Cincinnati, OH, USA) was added according to the manufacturer’s instructions (1 mL of reagent per 10^6^ cells). The samples were kept at −20 °C until RNA extraction.

#### 2.7.4. RNA Extraction and PCR Analysis

RNA was extracted from the goat PBMCs using RNAzol^®^ RT (Molecular Research Center Inc., Cincinnati, OH, USA) according to the manufacturer’s instructions. Contaminating chromosomal DNA was digested with DNase I (RNAse free; 1 IU/μg of RNA; Fermentas, Glen Burnie, MD, USA) for 30 min at 37 °C. RNA quality was assessed using an Agilent bioanalyzer total RNA nano chip. RNA at a concentration of 62.5 ng from each sample (per 10 µL reaction) was reverse transcribed and amplified by PCR using an iTaq™ Universal SYBR Green One-Step Kit (Bio-Rad, Hercules, CA, USA) and specific primers for the examined genes (see Table 1 for details). Primer sequences were designed using Oligo primer analysis software version 7.6 (Molecular Biology Insights, Inc., Colorado Springs, CO, USA) according to Gene Bank published sequences. Each primer pair was calibrated to determine the optimal reaction temperature and RNA concentration. Expression levels of examined genes were determined using RT-PCR. The RT-PCR was performed using a C1000 Thermal Cycler, and the results were analyzed using Bio-Rad’s CFX manager™ Maestro 2.3 software version 5.3.022.1030 (http://www.bio-rad.com/webroot/web/pdf/lsr/literature/10021337.pdf, accessed on 1 August 2024) (Bio-Rad, Hercules, CA, USA). Dissociation curve analysis was performed at the end of each real-time PCR reaction to validate the presence of a single-reaction product and lack of primer dimerization. Normalized expression (ΔΔC_q_) of examined genes was determined using two normalizing genes (goat18S and goat 28S). The calculation for normalized expression is described in the following formula, which uses the calculated relative quantity (RQ) calculation:

Normalized Expressionsample(GOI)=RQ sample (GOI)RQsampleRef 1×RQsampleRef 2×…×RQsampleRef n1n

where:

RQ = relative quantity of a sample

Ref = reference gene in a run that includes one or more reference genes in each sample

GOI = gene of interest (one target)

**Table 1:** Genes and primers sequences.

Sequence	Forward/Reverse	Gene Name	Gene Bank Code
5′GCAATTATTCCCCATGAACGAGG3′	F	Capra Hircus 18S	DQ149973.1
5′GGCAGGGACTTAATCAACGCAA3′	R		
5′GGCGAAAGACTAATCGAACCA3′	F	Capra Hircus 28S	AY894418.1
5′AGAGCGCCAGCTATCCTGA3′	R		
5′CTCCAGCCACAAACACTGACA3′	F	Capra Hircus IL-6	NM_001285640.1
5′ACCTTTGCGTTCTTTACCCAC3′	R		
5′GCAACCGTACCTGAACCCA3′	F	Capra Hircus IL-1β	DQ837160.1
5′GCCATCAGCCTCAAATAACAGC3′	R		
5′TGATGACTGCCCTGATCAAGC3′	F	Capra Hircus HSP70	JN604433.1
5′TACACCTGGATCAGCACACC3′	R		
5′CCAACCTGTGTCAACTGTGCAA3′	F	Capra Hircus Transferrin	GQ149766.1
5′TCCTTGACAAAAGCCACGTCT3′	R		
5′AGTTAATGCCTGTCACATACCCT3′	F	Capra Hircus Lysozyme	NM_001285711.1
5′CCATGCTCTAATGCCTTGTGGA3′	R		

### 2.8. Statistical Analysis

Statistical analyses and graphs were performed using JMP^®^ software version 17 (SAS^®^ Institute Inc., Cary, NC, USA). Sample size was calculated to allow significant statistical differences of at least 20%, with standard deviation less than 20% and a power of above 85%.

Prior to the analysis, the data were examined for normality to ensure parametric statistical analysis (ANOVA) could be performed. For serological tests, the data were analyzed using a two-way analysis of variance (ANOVA) to determine the significance of differences and the interactions between experimental treatments and physiological statuses with random blocks (yards), following Tukey-HSD test for more conservative multiple comparisons (for data with equal variances) or Steel–Dwass all pairs (for data with unequal variances), to determine the significance of differences between mean values. For gene expression tests, data were analyzed using a one-way analysis of variance (ANOVA) to assess the significance of differences between experimental treatments in each physiological status with random blocks (yards), following a Student’s *t*-test (for data with equal variances) or Welch–Student t for paired comparisons (for data with unequal variances), to determine the significance of differences between treatments mean values; comparisons were made between the experimental treatment and respective control. In all cases, values were considered significantly different, at the least, at *P* < 0.05.

## 3. Results

Stress is considered to perturb the animal’s body’s homeostasis. We first conducted a complete blood count (CBC) and blood biochemistry analysis to establish homeostasis in goats with or without environmental enrichments. Table 2 shows no significant differences in CBC values between the physiological groups and treatments. Moreover, all the parameters fall within the normal reference range.

Most of the measured biochemical parameters were also unaffected between groups and treatments (Table 3). However, changes were observed in the albumin, ALT, sodium, and chloride levels. Enrichment did not affect albumin levels, the major plasma protein, in dry goats. However, albumin levels were found to be higher, insignificantly so, in milking goats when compared to dry goats. The albumin concentration declined significantly during the enrichment treatment in milking goats. Also, the liver enzyme alanine transaminase (ALT) was significantly lower in milking goats than in dry goats. This difference became insignificant following the enrichment treatment due to an albumin increase in milking goats and a decrease in dry goats. Lastly, sodium and chloride levels were significantly higher in dry goats than in milking goats. The enrichment caused the decline in electrolyte levels in dry goats and the elevation of electrolyte levels in milking goats. This change in trend was significant in both dry and milking goats. Thus, the biochemical observations suggest that the environmental enrichment affected each goat’s group (dry vs. milking) differently.

**Figure 1:**
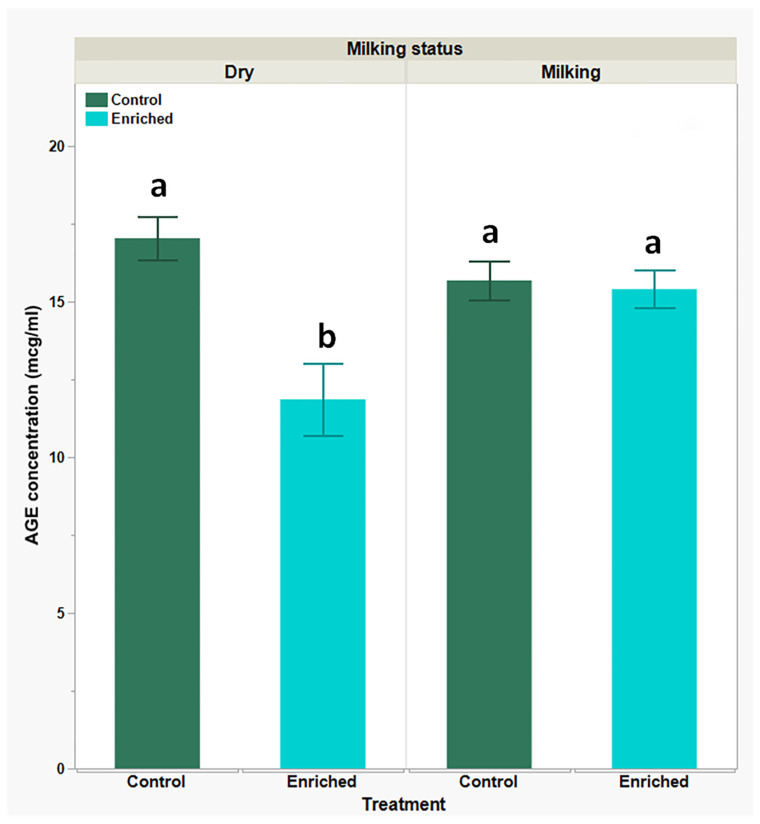
Serum AGE concentration in dry- and milking-goats groups prior to and following environmental enrichment; levels obtained using quantitative ELISA. Each bar represents mean (green or blue) ± SEM of 12 individual goats’ measurements. Two-way ANOVA model used to determine the significance of differences and the interactions between experimental treatments and physiological statuses, following Tukey-HSD test for multiple comparisons to determine significance of differences between mean values; means lacking a common superscript letter differ (*P* < 0.05).

Previous research published by us and others suggested that oxidative stress is a common outcome for many stressors (husbandry or others). Thus, AGEs are reactive metabolites of oxidative stress which may serve as a good marker for stress. To assess the impact of the environmental enrichment on the goats’ oxidative stress status, we determined AGE levels in the goats’ serum. The data in Figure 1 show very similar basal AGE serum levels in the control groups (dry versus milking, 17.03 ± 0.7 versus 15.67 ± 0.62 µg/mL, respectively). Notably, while environmental enrichment caused a substantial decrease in AGE levels in the dry-goats group (11.85 ± 1.17 µg/mL; *P* < 0.05), no effect was observed in the milking-goats group, and levels remained similar to the basal levels (15.4 ± 0.59 µg/mL).

**Figure 2:**
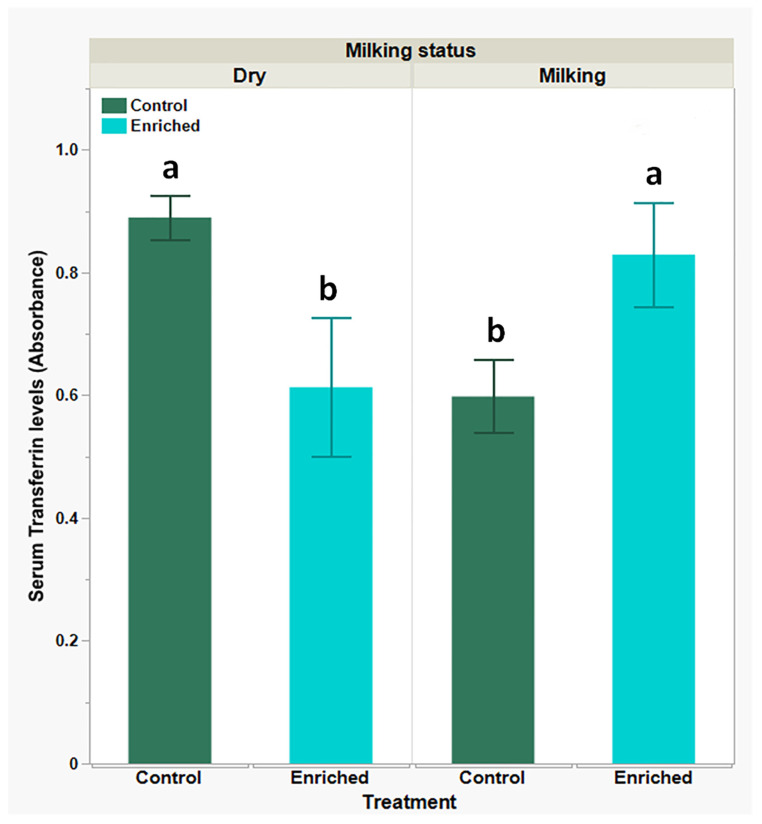
Serum transferrin levels in dry- and milking-goats groupsprior to and following environmental enrichment; levels obtained using indirect ELISA. Each bar represents mean (green or blue) ± SEM of 12 individual goats’ measurements. Two-way ANOVA model used to determine the significance of differences and the interactions between experimental treatments and physiological statuses, following Tukey-HSD test for multiple comparisons to determine significance of differences between mean values; means lacking a common superscript letter differ (*P* < 0.05).

Transferrin is an endogenous antioxidant known to bind and neutralize AGE. Consequently, we explored the effect of the enrichment treatment on serum transferrin levels. Results presented in Figure 2 show lower serum transferrin basal levels in the milking-goats group compared to the respective dry-goats group (*P* < 0.05). Transferrin levels changed oppositely following environmental enrichment: there was a significant (*P* < 0.05) decline in transferrin levels in the dry-goats group and a significant (*P* < 0.05) increase in transferrin levels in the milking-goats group (0.89 ± 0.03 EAU vs. 0.58 ± 0.12 EAU and 0.59 ± 0.05 EAU vs. 0.82 ± 0.08 EAU, respectively).

**Figure 3:**
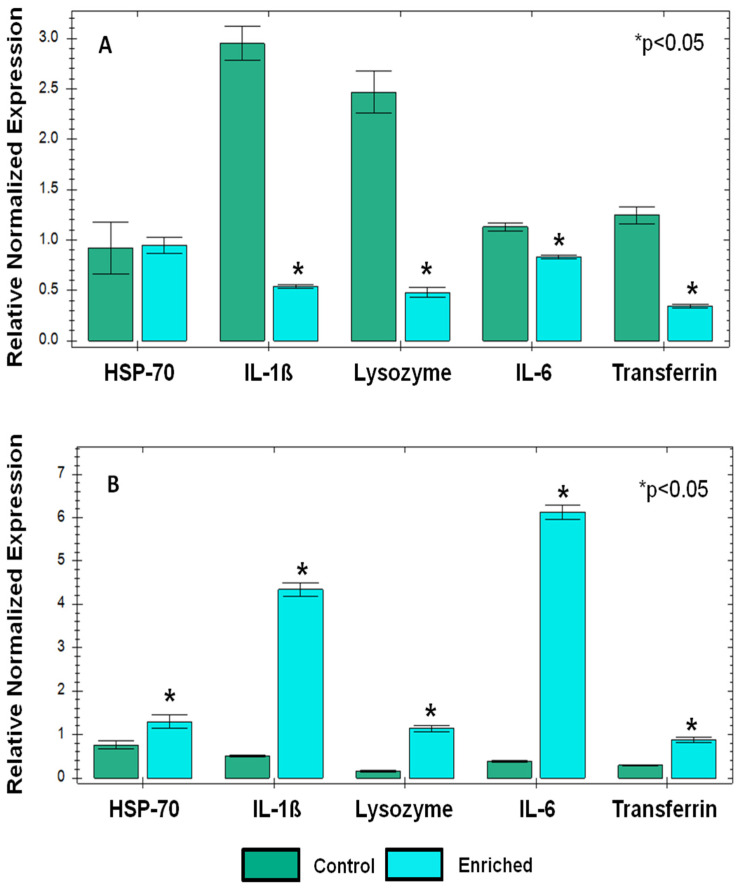
Gene expression of pro-inflammatory cytokines, stress-related, and anti-oxidant genes in PBLs prior to and following environmental enrichment in dry (panel (**A**)) and milking (panel (**B**)) goats groups. Genes’ relative fold expressions obtained using real-time PCR. For each investigated gene, each bar represents mean ± SEM of 12 individual goats’ measurements. For each physiological group (dry or milking), one-way ANOVA model was used to determine significance of differences between experimental treatments, following Student’s *t* test, to determine significance of differences between treatments mean values (*P* < 0.05); differences were marked using asterisks.

The immune system, specifically PBLs, is known to react to different stressors and to assist in restoring homeostasis. Following the changes observed in serum AGE and transferrin levels following the environmental enrichment in both physiological goat groups, we were prompted to determine stress-related and cytokine gene expression before and following the enrichment treatment in PBMC of dry and milking goats. Environmental enrichment in the dry-goats group led to several changes in gene expression (Figure 3A): The pro-inflammatory cytokines, IL-1ß and IL-6, expressions were significantly lower following enrichment (~6-fold and ~1.5-fold, respectively). Furthermore, after enrichment, gene expression of the immune-related antioxidants, lysozyme, and transferrin was also significantly lower (~5- and ~3-fold, respectively). Gene expression of the cell stress marker, heat shock protein 70 (HSP-70), was not altered. Interestingly, the data from the milking goats presented in Figure 3B showed a different trend (Figure 3B): a significant increase in HSP-70 expression (~1.5-fold), significant increases in pro-inflammatory cytokines, IL-1ß and IL-6, expression (~8-fold and ~12-fold, respectively), and a significant increase in the expression of the immune-related antioxidants lysozyme and transferrin (~4 fold). Moreover, a comparison between basal genes expression levels (control) of all the genes studied in dry- versus milking-goats groups revealed a significantly lower basal expression in the milking-goats group.

Lastly, the endocrine system is an important means of coping with stress. In this context, the two main anti-stress hormones, oxytocin and serotonin, can be affected by stress but also affect different physiological systems. Hence, we were prompted to assess their serum levels before and after the enrichment treatment.

**Figure 4:**
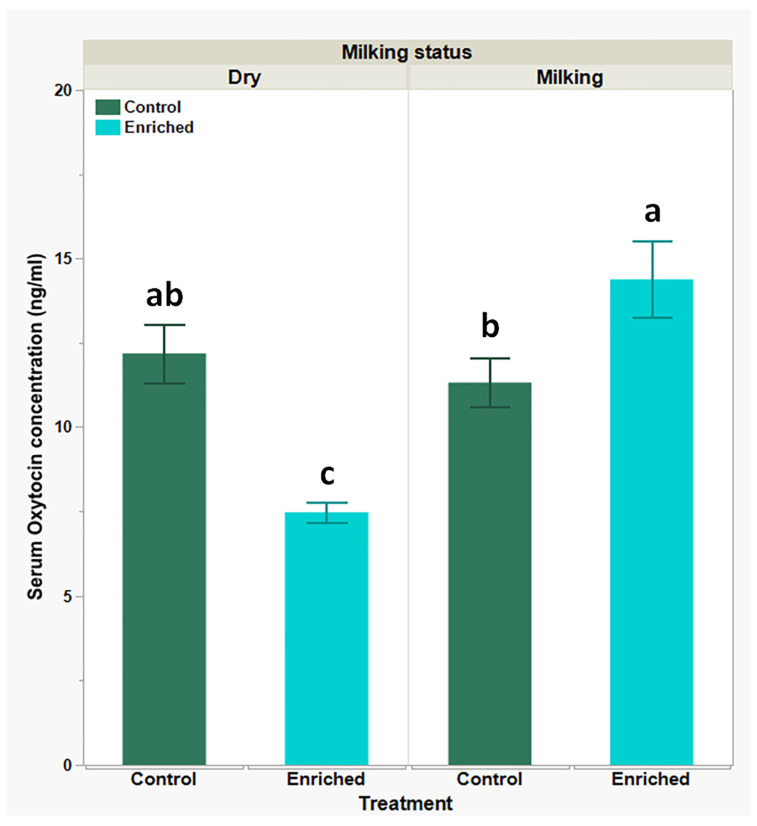
Serum oxytocin levels in dry- and milking-goats groups, prior to and following environmental enrichment; levels obtained using competitive ELISA. Each bar represents mean (green or blue) ± SEM of 12 individual goats’ measurements. Two-way ANOVA model used to determine the significance of differences and the interactions between experimental treatments and physiological statuses, following Tukey-HSD test for multiple comparisons to determine significance of differences between mean values; means lacking a common superscript letter differ (*P* < 0.05).

Data presented in Figure 4 show that serum oxytocin basal levels (control) were slightly higher in the dry-goats group when compared to the milking-goats group (12.18 ± 0.87 ng/mL and 11.32 ± 0.72 ng/mL, respectively), but following the enrichment treatment, there was a significant decline in the dry-goats group (7.47 ± 0.31 ng/mL) along with a significant increase in the milking-goats group (14.38 ± 1.14 ng/mL).

**Figure 5:**
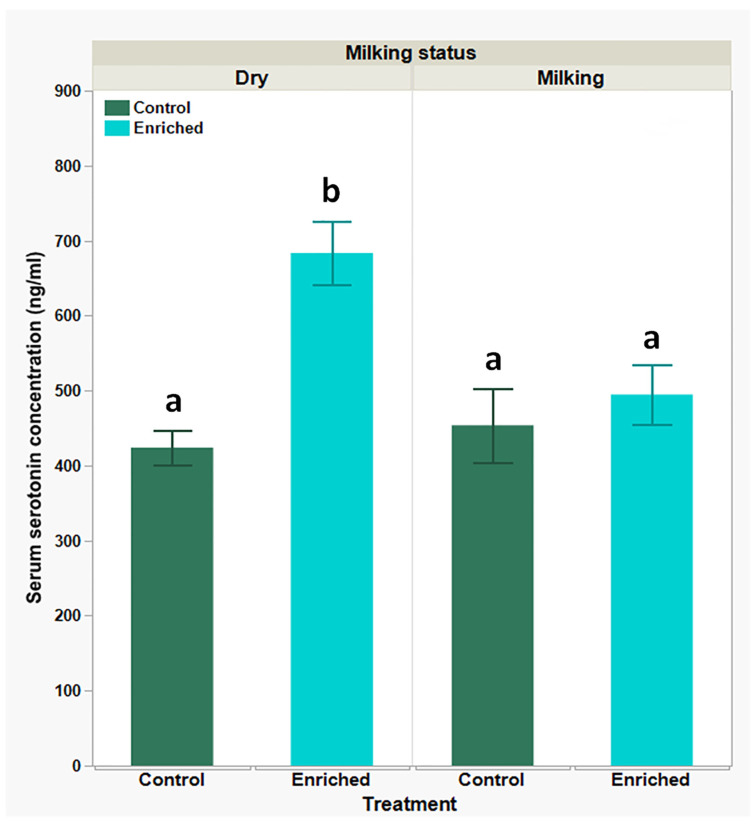
Serum serotonin levels in dry- and milking-goats groups, prior to and following environmental enrichment; levels obtained using competitive ELISA. Each bar represents mean (green or blue) ± SEM of 12 individual goats’ measurements. Two-way ANOVA model used to determine the significance of differences and the interactions between experimental treatments and physiological statuses, following Tukey-HSD test for multiple comparisons to determine significance of differences between mean values; means lacking a common superscript letter differ (*P* < 0.05).

A different trend was observed with serum serotonin levels (Figure 5): similar basal levels were measured in both the dry- and milking-goats groups (423.72 ± 23.5 ng/mL and 453.93 ± 49.34 ng/mL, respectively), with a marked and significant increase in levels of the dry-goats group (683.50 ± 42.54 ng/mL) and no significant change in the milking-goats group (494.76 ± 39.97 ng/mL) after the enrichment treatment.

## 4. Discussion

As homeostasis includes the equilibrium of many biochemical processes [35,36], it is quite understandable why the CBC and blood biochemical analysis are considered cost-effective and high-yielding tests to determine the animal’s homeostatic state [37,38].

In our current study, all hematological parameters were within normal reference ranges [38,39]. Differently, the blood biochemical analysis revealed a few interesting observations; while most parameters were also within the normal reference range [38], changes in the levels of albumin, ALT, sodium, and chloride were detected.

While clinically, the concentration of albumin (the major serum protein manufactured by the liver) can decline due to multiple reasons such as loss (e.g., protein-losing enteropathy or nephropathy, severe and extensive skin injury) or a failure in production, as occurs in liver failure or malnutrition, these reasons are very unlikely to explain our observations, as the goats in this study were closely monitored and did not suffer from any of the conditions mentioned above [40,41,42,43]. In addition, while data regarding albumin levels in milking versus dry goats are lacking, Prakash et al. found higher serum albumin levels in lactating cows compared to dry ones, supporting our observation regarding the difference between dry and milking control goats [44]. Yet, lactation does not explain the effect of the environmental enrichment in the milking-goats’ group. A more suitable explanation can be based on the rationale that the pro-inflammatory process, which characterizes many stress scenarios, and more specifically, the secretion of cytokines such as IL-6 and IL-1ß and tumor necrosis factor-alpha, were found to inhibit albumin levels [42,45,46]. Meaning: that elevated stress levels may cause a decline in albumin concentration and vice versa. Similarly, elevated liver enzyme ALT levels are commonly related to liver injury. Still, it has been repeatedly reported in post-stress scenarios in different animal species, particularly goats [38,47,48,49,50,51]. Likewise, reductions in plasma salt levels (low sodium and chloride levels) can occur due to many pathological reasons [52] but also due to stress-induced ADH/vasopressin release, following water re-absorption and consequently plasma dilution [52,53,54,55].

Thus, these observed changes may suggest an increase in stress levels in the milking goats following the environmental enrichment and higher basal stress levels in the dry control goats compared to the milking goats.

Interestingly, many publications suggest oxidative stress is a common result of many stress scenarios. Hence, oxidative stress markers can be sensitive and reliable for determining stress [56,57,58,59,60]. An example of such a marker is advanced glycation end products (AGE), which are reactive metabolites that can be formed due to lipids peroxidation, a process that lies at the core of response to a broad spectrum of stressors [30,61,62,63,64,65,66,67]. Accordingly, our data suggest that while the environmental enrichment treatment managed to decrease stress in the dry-goats group (manifested in low AGE serum levels), it did not affect the milking-goats group or it putatively induced stress *de novo*, which was neutralized by the goats’ physiological buffering systems (manifested in unchanged AGE serum levels).

A good example of such a buffering system is the endogenous immune-related antioxidant reagent transferrin. While its primary function in the body is iron binding and transportation throughout tissues (thus, protecting them from the deleterious oxidative effect of free iron ions), it was found to perform as an antioxidant by binding AGE and neutralizing it in addition to its other immunologic properties such as iron depravation from bacteria [30,68,69,70,71,72]. Hence, we expect serum transferrin levels to be reciprocal to an oxidative stress burden: increase during oxidative stress and decrease when it lessens. In practice, our recorded data support this claim and further suggest that the basal oxidative burden is higher in the dry-goats group (compared to the milking-goats group). Although the environmental enrichment reduced stress in the dry-goats group, it induced *de novo* stress in the milking-goats group. Moreover, elevated serum transferrin levels with unchanged serum AGE levels were more likely due to transferrin’s ability (and other endogenous antioxidants such as serum lysozyme) to neutralize AGE rather than no treatment effect [73,74].

The immune system is a vital, valuable, and available resource to be used by the animal’s body to alleviate stress and protect the body’s tissues from additional damage. In this context, two main mechanisms are well known: the first is the previously discussed facilitation of endogenous immune-related antioxidants, which can interact and neutralize oxidative metabolites that may further perturb homeostasis and elicit further damage to the already disturbed physiological status, and the second is the activation of the pro-inflammatory immune response to increase alertness versus opportunist pathogens that may try to exploit this susceptible situation and to inflict further damage to the animal’s body [20,21,50,75]; that is in addition to its other cardinal role in inducing inflammation for the purpose of tissue amendment [29,76,77,78]. Interestingly, as the immune system facilitates oxidative stress to fight pathogens, its activation may induce further production of anti-oxidants, suggesting the constant interplay between anti-oxidants and pro-inflammatory cytokines [79,80]. Additionally, our previous work and that of others demonstrated that the blood tissue and, more specifically, peripheral blood leukocytes (PBLs) are in close interaction with almost every other tissue in the body and respond to stress (among other functions) by up-regulating the expression of pro-inflammatory cytokines such as IL-1ß and IL-6, stress-related genes such as HSP-70, and endogenous immune-related anti-oxidants: transferrin and lysozyme. This makes them suitable biosensors for stress [20,21,22,23,29,30,81,82,83].

Similarly to the trends seen in the serum AGE and transferrin analysis, the gene expression analysis revealed that the enrichment treatment induced down-regulation in the expression of four out of five target genes (two pro-inflammatory and two antioxidant genes) in the dry-goats group. In contrast, it induced up-regulation of all target genes in the milking-goats group. Furthermore, the basal expression of these genes was lower in the milking-goats group. Taking into consideration the discussed interplay between anti-oxidants and pro-inflammatory cytokines, it is strongly suggested that the enrichment treatment in the milking-goats group was translated into a danger signal (oxidative stress), to which the immune response reacted by up-regulating pro-inflammatory cytokines and anti-oxidants genes expression and vice versa in the dry-goats group, meaning that the declined stress signal, down regulated the same target genes.

Finally, although the endocrine system is more commonly mentioned for its ability to utilize other physiological systems to cope with stress, it can also be affected by stress; hence, its components could serve as stress indicators [1,2,84,85]. The two anti-stress hormones, oxytocin and serotonin, should be mentioned in this context.

Oxytocin is best known for its physiological role in milk production and uterus contraction during parturition in many mammals [86]. However, additional functions were attributed to it in different species. Among these functions, behavioral and olfactory conditioning, such as maternal behavior (rat, mouse, and human), bonding formation (vole, sheep), social cognition (mouse, human), anxiety relief (mouse, rat, and human), eye contact and trust (human), individual cognition (mouse, sheep) and physiological functions such as pain relief (mouse, rat, and human), stress alleviation (rat, sheep, and human), along with anti-inflammatory, anti-apoptotic, and anti-oxidative effects in vast physiological systems, such as immune, nervous and cardiovascular, digestive, musculoskeletal and renal, metabolic, and respiratory, respectively [86,87,88,89,90]. Moreover, its secretion was found to be mostly social via olfactory, auditory, visual, and physical stimuli [89,90].

As such, it is suggested that oxytocin can serve as a more specific marker for social stress, meaning that oxytocin levels will rise following stress. In its absence, oxytocin levels will drop. This claim is consistent with our current observations. Differently, serotonin, although considered an anti-stress hormone that promotes good mood and well-being (due to its other physiological functions), is more susceptible to stress, which tends to inhibit its production and secretion. This often translates into increased anxiety and fear [91,92,93]. Though the increase in serotonin levels seen in the dry-goats group following the enrichment treatment meets with this consensus and our prior observations, the unchanged levels in the milking-goat group following the enrichment treatment are not. We propose that this is more likely due to low sensitivity or interplay with other hormones, such as oxytocin and cortisol, rather than a no treatment effect [93,94]. Another possible explanation for this observation, is that differently from oxytocin, which is mainly produced in the hypothalamus and secreted from the pituitary [95], serotonin’s main serum’s origin is peripheral to the nervous system (platelets and enterochromaffin cells within the intestine), suggesting serotonin serum levels can vary, due to other non-stress related physiological processes (local or systemic, such as platelets metabolism) [92,93].

## 5. Conclusions

In conclusion, the presented body of observations suggests that environmental enrichment (stages and brushes), which was used in our current research oppositely affected the goats (depending on their physiological statuses), alleviated stress in the dry goats while putatively inducing *de novo* stress in the milking goats, and this could be due to different physical and social needs, required by each physiological phenotype. Unsurprisingly, evidence for the deleterious effect of environmental enrichment (as suggested in the milking-goat group) has already been documented in different species [96,97,98]. Interestingly, the basal stress levels in the milking goats seem to be lower (compared to the dry goats); we suggest this could be due to their daily milking routine, facilitating the release of oxytocin [99,100], which possesses the qualities discussed above and more specifically the anti-inflammatory and anti-oxidative ones. Nevertheless, it is important to mention the limitations of this study: mainly, it was performed in a single, and only one enrichment factor (brushes and stages) was used. Moreover, although given 30 days of adaptation, goats were sampled post 10 days of treatment (enrichment or control), which means that more chronic responses to the enrichment were not recorded in the investigated time frame. Concurrently, we recommend continuing to research other means to alleviate stress in farm animals, using the described methodology while keeping in mind that animals with different physiological statuses can react differently.

## Figures and Tables

**Table 2:** CBC hematological values (Mean ± SEM).

Parameter	Dry Goats Mean ± SEM		Milking Goats Mean ± SEM		*P*-Value
	Control	Enriched	Control	Enriched	
WBC (10^3^/µL)	8.63 ± 1.13	8.34 ± 0.86	9.13 ± 0.96	9.67 ± 0.86	0.6683
RBC (10^6^/µL)	14.80 ± 0.56	15.24 ± 0.45	14.86 ± 0.63	14.48 ± 0.55	0.4677
HGB (g/dL)	9.19 ± 0.31	9.62 ± 0.24	9.40 ± 0.37	9.14 ± 0.28	0.2589
HCT (%)	24.96 ± 0.9	26.01 ± 0.7	25.13 ± 1	24.35 ± 0.81	0.2999
MCV (fl)	16.9 ± 0.29	17.1 ± 0.3	16.99 ± 0.48	16.93 ± 0.52	0.7623
MCH (pg)	6.22 ± 0.10	6.33 ± 0.10	6.44 ± 0.18	6.36 ± 0.18	0.5588
MCHC (g/dL)	36.91 ± 0.20	37.03 ± 0.15	37.44 ± 0.28	37.57 ± 0.23	0.9944
RDW (%)	26.37 ± 0.51	26.23 ± 0.42	26.30 ± 0.5	26.33 ± 0.25	0.8538
PLT (10^3^/µL)	260 ± 37.59	287.09 ± 43.72	309.82 ± 61.53	243.5 ± 44.59	0.3411
MPV (fl)	9.93 ± 0.62	9.98 ± 0.59	9.86 ± 0.77	11.5 ± 0.73	0.2590
Neut (10^3^/µL)	3.98 ± 0.52	3.22 ± 0.32	3.18 ± 0.51	3.83 ± 0.35	0.1211
Lymph (10^3^/µL)	4.27 ± 0.51	4.59 ± 0.6	5.45 ± 0.71	5.34 ± 0.70	0.7384
Mono (10^3^/µL)	0.13 ± 0.02	0.12 ± 0.02	0.12 ± 0.02	0.13 ± 0.01	0.6146
Eos (10^3^/µL)	0.44 ± 0.1	0.34 ± 0.09	0.19 ± 0.08	0.27 ± 0.08	0.2985
Baso (10^3^/µL)	0.03 ± 0	0.03 ± 0	0.05 ± 0.01	0.05 ± 0	0.7115
LUC (10^3^/µL)	0.01 ± 0	0.01 ± 0	0.02 ± 0	0.03 ± 0	0.1692
Neut (%)	42 ± 3.1	39.84 ± 2.77	35.53 ± 4.44	40.85 ± 3.12	0.2872
Lymph (%)	46.89 ± 5.03	54.21 ± 3	54.90 ± 5.94	53.86 ± 2.89	0.3526
Mono (%)	1.50 ± 0.29	1.64 ± 0.33	1.27 ± 0.21	1.50 ± 0.23	0.8528
Eos (%)	3.83 ± 0.56	3.64 ± 0.51	1.98 ± 0.49	2.12 ± 0.51	0.7528
Baso (%)	0.40 ± 0.04	0.4 ± 0.05	0.56 ± 0.08	0.57 ± 0.04	0.8875
LUC (%)	0.17 ± 0.02	0.25 ± 0.47	0.37 ± 0.10	0.43 ± 0.17	0.9850

**Table 3:** Blood biochemistry analysis (mean ± SEM).

Parameter	Dry Goats Mean ± SEM		Milking Goats Mean ± SEM		*P*-Value
	Control	Enriched	Control	Enriched	
CK (U/L)	151.45 ± 10.92	133.63 ± 10.92	155.25 ± 10.46	145.08 ± 10.46	0.7224
Albumin (g/dL)	3.30 ± 0.13 ^ab^	3.56 ± 0.16 ^ab^	3.70 ± 0.19 ^a^	3.10 ± 0.10 ^b^	* 0.0325
ALKP (U/L)	344.45 ± 88.14	319.45 ± 83.04	192.66 ± 56.09	203.41 ± 54.4	0.8020
ALT (U/L)	16.50 ± 0.58 ^a^	14.34 ± 0.72 ^ab^	13.12 ± 0.33 ^b^	15.51 ± 0.44 ^ab^	* 0.0030
SuperAMY (U/L)	17.1 ± 3.82	19.27 ± 3.58	18.66 ± 2.24	19.41 ± 2.40	0.8145
AST (U/L)	62.55 ± 3.51	57.66 ± 2.80	64.98 ± 2.70	66.4 ± 2.85	0.2810
Total bile (mg/dL)	0.03 ± 0.02	0.05 ± 0.01	0.04 ± 0.02	0.06 ± 0.02	0.9578
Calcium (mg/dL)	8.41 ± 0.15	8.57 ± 0.15	8.49 ± 0.16	8.15 ± 0.18	0.1380
Cholesterol (mg/dL)	85.93 ± 3.86	85.15 ± 4.34	92.73 ± 4.20	89.45 ± 4.80	0.7761
Creatinine (mg/dL)	0.74 ± 0.04	0.76 ± 0.04	0.78 ± 0.03	0.74 ± 0.04	0.5354
GGT (U/L)	51.63 ± 4.49	54.58 ± 4.3	60.08 ± 4.3	54.58 ± 4.30	0.3347
Glucose (mg/dL)	43.92 ± 2.85	38.07 ± 2.84	41 ± 3.16	44.25 ± 1.98	0.1061
Phosphate (mg/dL)	6.66 ± 0.45	6.36 ± 0.50	7.27 ± 0.31	7.68 ± 0.44	0.4285
Total protein (g/dL)	7.59 ± 0.12	7.70 ± 0.11	7.52 ± 0.08	7.41 ± 0.11	0.3289
Triglycerides (g/dL)	25.27 ± 3.22	24.78 ± 2.27	19.37 ± 2.53	24.98 ± 2.94	0.2795
Urea (mg/dL)	37.51 ± 3.53	37.56 ± 3.38	43.35 ± 2.01	46.17 ± 1.58	0.6087
Sodium (mmol/L)	146.36 ± 0.60 ^b^	148.5 ± 0.47 ^a^	149.75 ± 0.55 ^a^	146.4 ± 0.46 ^b^	* <0.0001
Potassium (mmol/L)	4.89 ± 0.11	4.79 ± 0.10	4.92 ± 0.13	4.92 ± 0.11	0.3763
Chloride (mmol/L)	104.47 ± 0.54 ^b^	107.13 ± 0.93 ^a^	107.24 ± 0.97 ^a^	103.35 ± 0.66 ^b^	* <0.0001

Two-way ANOVA model used to determine the significance of differences and the interactions between experimental treatments and physiological statuses; model statistical significance (*P* < 0.05) is marked by asterisks. Following Tukey-HSD test for multiple comparisons to determine significance of differences between mean values; means lacking a common superscript letter differ (*P* < 0.05).

## Data Availability

The original contributions presented in the study are included in the article/Supplementary Materials, further inquiries can be directed to the corresponding authors.

## Supplementary Materials

The following supporting information can be downloaded at: https://www.mdpi.com/article/10.3390/biology13110859/s1, Figure S1: Photo describing the environmental enrichment, used in the current research.
