# Supplementary material for: The Effect of Environmental Enrichment on Selected Physiological and Immunological Stress-Related Markers in Dairy Goats
Source: Biology (Basel). 2024 Oct 24;13(11):859. doi: 10.3390/biology13110859 (PMC11591861; doi:10.3390/biology13110859)

Supplementary Materials:

Figure S1: Photo describing the enviormental enrichment, used in the currer reserch. As described in the material and methods section, environmental enrichment was in the form of free access to an in-yard installed static scratch brush (length 80mm; diameter 40mm with the ability to swing from east to west and north to south at a 270° angle [Melasty®, Nilüfer/Bursa, Türkey]), and a wooden stage (custom made from oak wood: 120×120 mm^2 face area raised on wooden legs 80 mm high).

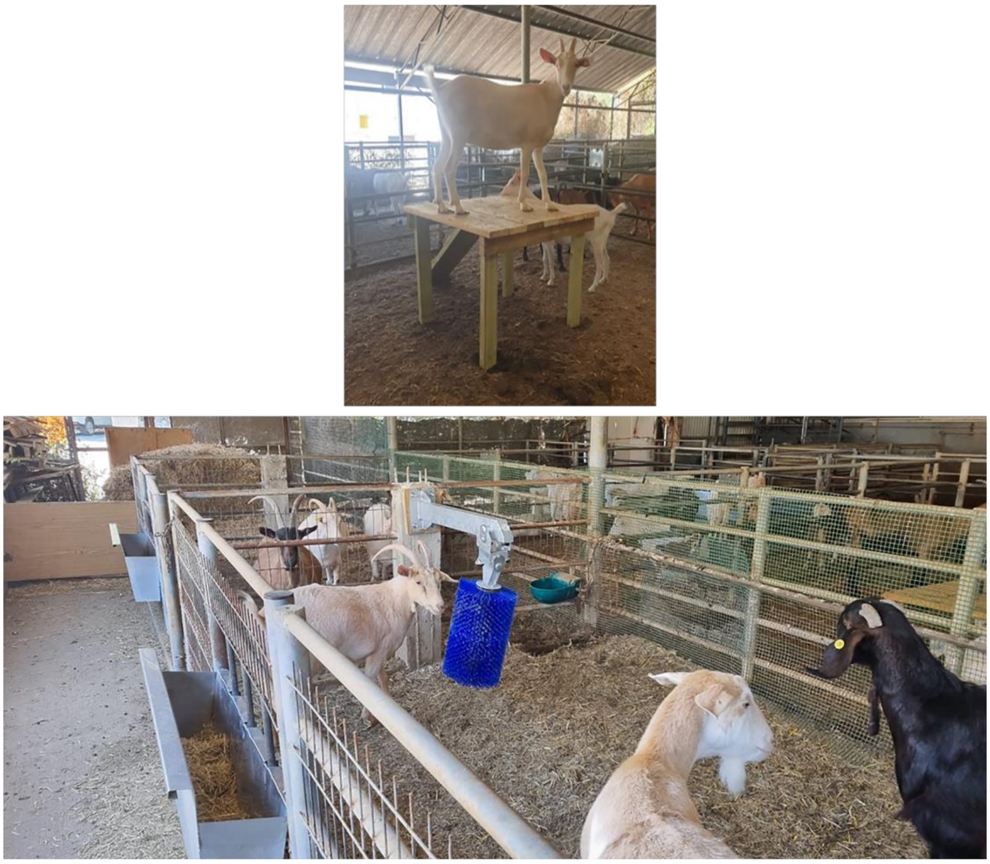

Supplement: Supplementary file 1 [file biology-13-00859-s001.zip › biology-3250288-supplementary.pdf]
